# Moments that matter: childhood pain treatment shapes pain for life—we can do better every time in every child

**DOI:** 10.1186/s12916-025-03869-7

**Published:** 2025-02-04

**Authors:** Rebeccah Slater, Suellen Walker, Christopher Eccleston, Carlo Bellieni, Tanvi Hirekodi, Ricardo Carbajal, Lucinda Smart, William Laughey, Maria M. Cobo, Stefan Friedrichsdorf

**Affiliations:** 1 Department of Paediatrics, University of Oxford, Oxford, UK; 2 Developmental Neurosciences Department, UCL GOS Institute of Child Health, UCL, London, UK; 3 Centre for Pain Research, The University of Bath, Bath, UK; 4 Department of Experimental, Clinical and Health Psychology, Ghent University, Ghent, Belgium; 5 Department of Psychology, The University of Helsinki, Helsinki, Finland; 6 Department of Pediatrics, University of Siena, Siena, Italy; 7 School of Medicine, Newcastle University, Newcastle Upon Tyne, UK; 8 Pediatric Emergency Department, Assistance Publique-Hôpitaux de Paris, Hôpital Armand Trousseau- Sorbonne Université, INSERM U1153 Paris, France; 9 Reckitt Health Care UK Ltd, Hull, UK; 10 Health Professions Education Unit, Hull York Medical School, York, UK; 11 Colegio de Ciencias Biologicas y Ambientales, Universidad San Francisco de Quito USFQ, Quito, Ecuador; 12 Division of Pediatric Pain, Palliative Care & Integrative Medicine, University of California San Francisco, San Francisco, USA

**Keywords:** Children, Infant, Pain, Needle procedures, Pain treatment, Information, Motivation, Communication, Parents, Healthcare professionals

## Abstract

**Background:**

Needle procedures, such as vaccinations, blood draws, and intravenous cannulation, are the most frequent source of childhood pain, causing fear and reducing the uptake of medical procedures. Every child has the right to expect pain relief, and we have evidence-based tools to reduce needle procedure-related pain. Therefore, the lack of analgesic provision for needle pain is not justified. We argue that better informed and motivated healthcare professionals and families can advocate for appropriate pain relief in every child, every time.

**Observations:**

Engaging communication campaigns are needed to educate our healthcare professionals. Evidence-based modalities such as topical anaesthesia, sucrose or breastfeeding, comfort positioning, and age-appropriate distractions should be available for every child during needle procedures. However, high-quality information is not enough to change behaviour—healthcare professionals need to be motivated, encouraged, and inspired. Parents and carers should be empowered to advocate for their children and be aware that their child has the right to receive pain relief during these procedures.

**Conclusions and relevance:**

This is a call to action—we need collaboration between academics, healthcare professionals, industry and charities, to expedite behavioural change and parental advocacy through high-quality communication strategies. Effective pain management in infants and children can play a crucial role in promoting the uptake of vaccinations and medical procedures and can influence future attitudes to pain.

## Background

### Pain in infants and young children matters

Children regularly experience needle-related procedures, such as vaccinations, blood draws, and intravenous cannulation. In the United Kingdom (UK), for example, from birth to 5 years of age, children receive 12 separate intramuscular injections to protect against serious diseases [1]. These procedures are the number one source of pain in childhood, and concern about pain is a major barrier to uptake [2, 3]. We know that untreated pain causes short-term distress that negatively impacts child and parental wellbeing [4] and shapes future attitudes to pain [5]. Given we have the evidence-based tools to reduce needle-related pain in *every child, every time* [6], untreated pain is unjustifiable.

In this article, we use needle-related pain to highlight three concepts that are key to driving positive change in the treatment of childhood pain. We describe (i) how high-quality information for healthcare professionals and parents is needed to ensure effective pain management and to debunk myths, (ii) that healthcare professionals will prioritise childhood pain treatment if they are motivated to do so, and (iii) that children need advocates to ensure that their pain is considered and treated.

### Untreated needle pain and needle fear can have profound consequences

The World Health Organisation (WHO) guidelines (2015) [7] state that the lack of analgesic provision for vaccination pain is inexcusable. Yet, pain caused by ‘minor’ procedures can all-too-frequently be dismissed as inconsequential [8]—ignoring the fact that when repeated over weeks, months and years, each momentary pain experience is compounded [9] and highly traumatic. What can be presented as ‘just one poke’ can have profound and lasting consequences on personal and public health [5]. For example, our co-author who experienced childhood bone cancer articulately describes how healthcare professionals can trivialise or dismiss pain caused by routine medical procedures because it is assumed that her early life trauma means she is more able to tolerate pain [8]—this is not true and can negatively impact the clinician-patient alliance.

A serious consequence of untreated vaccination pain is the establishment or exacerbation of needle fear, where 20–50% of adolescents report fear of needles [10]. Needle fear can delay or prevent the uptake of essential medical procedures [11–13] and is a leading cause of vaccine hesitancy in ~ 20% of the under-vaccinated population [14]. In the UK, while the number of vaccinations offered to children is increasing, uptake of vaccinations is decreasing [15]. This trend is concerning, especially considering that children from lower socio-economic groups, Black and Asian communities, and girls are less likely to be vaccinated [16, 17]. The consequences of falling vaccination rates can be profound, as exemplified by the recent UK measles crisis, in which measles vaccination has now dropped below the 95% needed to protect the population [18]. A lack of vaccine uptake for measles has resulted in unvaccinated symptomatic children and their siblings being told they cannot attend school for up to 21 days and cannot have contact with vulnerable people [19], despite the dire consequences of these actions, already exposed during the COVID-19 pandemic [20]. If pain caused by vaccination prevents a single family from vaccinating their children, this is difficult to defend, as we have the tools to provide effective pain relief [6]. The regular contact between healthcare professionals, children, and their families during routine vaccinations provides an opportunity to demonstrate best practices to manage pain and reduce needle-related fear. Furthermore, acknowledging and appropriately treating pain enables children to be actively involved in their care, gives them a sense of control, creates better memories of their experiences, and provides them with skills to cope with future pain [11, 12, 21].

### We can do better every time in every child

Parents are dissatisfied with their experience of childhood vaccinations: of 1485 parents, as many as 32% were dissatisfied with the information they received, and 27% were concerned about pain, distress, or side effects caused by the vaccination [22]. It is troubling, yet not surprising, that pain and distress are often cited as a primary parental concern [23]. A recent survey of 255 UK practising nurses (where the majority had > 10 years’ experience administering 5–20 childhood immunisations per week) highlights that only 13% received pain management training and that pain management policies were in place in < 5% of practices. Consequently, pain-relieving techniques are infrequently used; a consistent approach to *always* using topical local anaesthetics (0% of practices), sucrose (0%), breast-feeding (6%), or parent (6%) and nurse-led (13%) distraction techniques does not happen or is extremely rare. Worryingly, a frequent reason cited for not providing pain relief was that ‘parents didn’t request it’ [23]. How can parents request analgesia, if they were not told that it is their child’s right to expect pain relief during routine medical procedures? The data described here is not consistent with the ‘Comfort Promise’, which is an international initiative where participating institutions ‘*promise to do everything possible to prevent and treat needle pain. For every child. Every time*’ [24, 25]*.* Given we have evidence-based tools to treat pain caused by intramuscular injections as well as other procedures, why does it remain under-treated in children? What are the barriers to implementation? And how do we motivate healthcare professionals and families to expect this level of care? This article addresses these questions and identifies solutions.

### High-quality empirical evidence is important, but information provision is not enough

Providing pain relief in infants and young children is challenging (Table 1). Pain is a complex multifaceted individual experience that is difficult to describe [26]. Fundamentally, pain is protective: it signals damage to an individual, it provides a mechanism to promote healing, it drives compassionate care in others, and it educates us on how to protect our bodies. When children undergo painful necessary invasive procedures, pain can be perceived as a mechanism for future protection. This complexity makes it difficult for parents to know how to respond to induced pain in the healthcare setting. Expectations that children should ‘man-up’, ‘cope’, or ‘be brave’ are confusing. This conceptual confusion about the value of pain (by both parents and healthcare professionals) can be reduced through the provision of high-quality, accessible material that describes best evidence-based practice. Consensus-finding within this author-group identified key facts that need to be communicated to healthcare professionals and parents (Table 2). While these key aspects have been identified, further attention needs to be given to how this information is presented and delivered.

**Table 1:** Addressing the challenge of providing pain relief for infants and young children

The Problem:
• Pain is a complex phenomenon, which is difficult to measure in young children. This can lead to misunderstanding and false beliefs
• While preventing and treating pain in children should be everybody’s problem, institutionally, it is nobody’s responsibility, and, in most places, clinicians receive very little training or support to implement evidence-based analgesic strategies
• Treating pain can feel like a secondary action—i.e. it is considered less important than the underlying problem or task being performed (e.g. the vaccine being administered or the dental treatment conducted)
• In certain healthcare environments, pain in children is still considered inevitable and a normal consequence of care
• Parental beliefs and actions can substantially influence the degree of pain relief provided to children
• There can be a lack of personal accountability (i.e. consequences) if pain in infants and young children is not treated—unless the child has a strong advocate
• The repeated practice of administering painful procedures can accustom caregivers to children's pain reactions and reduce awareness of the pain being caused
• There is limited understanding of the efficacy of psychological treatments, leading to de-prioritisation of these approaches compared with pharmacological interventions. These approaches are viewed as ‘less medical’ or more ‘alternative’, and so are often considered less effective, despite evidence to the contrary
The Solution:
• New approaches to generate greater motivation and engagement around our ‘responsibility’ to prevent and treat pain in infants and young children is required. This can be through stories, art, and other powerful media
• Educating and demonstrating best practice to parents and carers will drive parental advocacy and ultimately, personal accountability
• Changing the perceived hierarchy that psychological interventions are less effective than pharmacological analgesics in infants and young children needs to be readdressed through careful messaging
• Upfront acknowledgement of our personal discomfort when we cause (iatrogenic) pain in children needs to be addressed, rather than dismissed or ignored

**Table 2:** Demystifying childhood pain: key facts that need to be communicated to healthcare professionals and parents

• Parents have the right to ask for and then receive evidence-based modalities for needle procedures for every child every time including (i) topical anaesthesia (e.g. lidocaine cream for at least 30 min), (ii) sucrose or breastfeeding for infants < 12 months, (iii) comfort positioning (e.g. swaddling or skin-to-skin care for infants < 6 months, sitting upright and never being held down for children > 6 months), and (iv) age-appropriate distractions (e.g. bubbles, pinwheel, apps) [24]
• Clinicians should not decline requests for pain relief by responding with debunked myths (reasons such as topical local anaesthetic takes too long to apply, it will cause vasoconstriction, and babies will associate pain with sucrose/breastfeeding if offered during painful procedures are not true)
• Inadequate analgesia for initial painful procedures in children diminishes the effect of adequate analgesia in subsequent procedures [27]
• Signs and consequences of pain, especially in infants and young children, can be indirect and complex and can outlast the duration of the experience
• Guidance about psychological interventions needs to be precise—with a similar style of information as would be provided for pharmacological interventions (e.g. providing dose, frequency, route of administration information)
• Children’s psychological framing of a painful experience can be positively influenced by parents and healthcare professionals [28], leading to reduced analgesic requirements in the future
• Babies remember pain. Boys circumcised without analgesia cry longer and harder at their 4–6-month vaccinations than boys who received circumcision analgesia [29, 30] and exposure to neonatal pain in premature infants is related to higher pain self-ratings during venepuncture at school age [31]

A key feature of successful implementation is that high-quality information provision is not enough to change behaviour—people (be they parents or healthcare professionals) do not act on information alone; they need to be motivated, encouraged, and inspired [32] to ensure that pain treatment is prioritised in busy healthcare settings and within the home environment. There are several factors, highlighted in Table 1, to explain why the treatment of pain in infants and young children is challenging. To overcome these challenges, we need to find ways to create greater awareness and engagement about the importance of alleviating pain in infants and young children. This should include the use of stories [33], art [34], and other media [35]—in contrast, in the context of post-operative pain, the way in which multiple warning signs about the potential adverse effects of pain relief are presented in leaflets does not encourage the appropriate use of pain relief [36]. Bringing childhood pain treatment to the top of the priority list requires novel and creative ways to engage with multiple stakeholders. For example, powerful infographics about the healthcare economic value of treating pain in young children should be shared with those responsible for resource allocation (e.g. healthcare leaders). Modern approaches (e.g. apps, social media platforms) provide benefits beyond printed material, but careful consideration of the audiences is required for bespoke information provision (e.g. appropriate language use, level of complexity and detail). Expertly produced materials using high-end communication strategies are required to promote these efforts. As an example, expert communication on hand-washing, which can lead to a reduction in transmission of germs, generated 75 million user videos on TikTok, with over 100 billion views in total [37, 38]. Similar high-quality communication and implementation strategies are needed to motivate healthcare professionals and parents to understand that pain caused by vaccination is unacceptable. There are excellent examples to follow [6, 39, 40], and consensus among healthcare providers has led to the successful introduction of pain-relieving strategies for vaccination [41]. Simply encouraging parental contact and reassurance during vaccinations, using age-appropriate distraction techniques, or reframing the experience post-vaccination could have a profound impact on vaccine uptake, which in turn would reduce viral transmission. In the context of the current measles outbreak, in the UK and elsewhere, a small increase in the number of individuals vaccinated can have dramatic positive effects [42].

### Parents can advocate for their children but need support as well

**Fig. 1:**
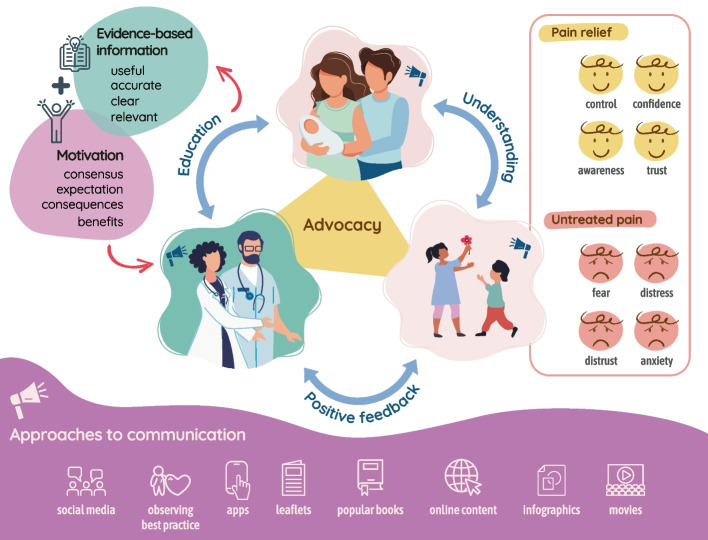
Highlights how coupling evidence-based information and motivational drive, in high-quality communications, can prevent avoidable pain in all children. Better informed and more knowledgeable healthcare professionals, families, and children can advocate for appropriate pain relief. This will promote positive emotional experiences during necessary painful procedures and prevent the negative sequalae associated with untreated pain

If pain caused by vaccine administration was eliminated, using well-established, evidence-based approaches [43], then procedure-related fear would likely diminish. With appropriate resources and time, we can make this a reality as we have tools such as local anaesthetic cream, breastfeeding, skin-to-skin care, comfort positions, distractions, and memory-shaping to reduce this type of pain [24, 43–49]. Nevertheless, it is important that parents act as their child’s advocates, with younger children potentially requiring greater advocacy than older children, given the difficulty in measuring pain in pre-verbal children. Therefore, parents need to be well-informed about the value of pain treatment as well as the potential consequences of untreated childhood pain [9, 10, 12]. Through this, they will recognise the importance of advocating for effective pain relief for their child. During vaccinations, parents frequently observe events such as children being held down, running away, screaming, or crying [12]. Observing these behaviours normalises them to parents and gives the perception that it is appropriate to manage painful procedures in this way. This can have a negative effect on parents’ and children’s attitudes to pain. Parents are empowered by knowledge and through observing examples of best practice. Figure 1 shows how dissemination of high-quality materials across various communication channels to parents, children, and healthcare professionals can encourage informed advocacy.

It may be easy to assume that the need for advocacy is unfounded given current understanding of best pain management practice, but several factors can undermine the best of intentions: (i) stress and time pressure in both healthcare and home environments, (ii) a focus on procedures (e.g. prioritising the administration of the vaccine over the importance of pain relief), (iii) a lack of resources and (iv) the needs of everyday life (e.g. the importance of getting back to work, school or home). In a recent research study, conducted at the John Radcliffe Hospital in Oxford, parents were offered topical anaesthetic for their babies prior to insertion of a cannula to test for jaundice [50], yet the uptake was low. The primary reason cited by parents was that they ‘didn’t want to wait for the local anaesthetic to work’ (personal communication).

Infants and young children have a right to receive appropriate pain prevention and treatment, and societal awareness, empowerment, and regulation are needed to make this a reality. Perhaps most importantly, we need to consider who stands with the parents in these challenging situations. Healthcare providers, with a holistic understanding of child and family needs, should take each opportunity to demonstrate best practice. By providing thoughtful guidance and information, they can enable parents to develop the expectation that no child will experience avoidable pain and distress in a healthcare setting (Table 3).

**Table 3:** Factors influencing the need for increased drive to support parental advocacy

• Parents expect pain to be relieved [51]
• Parents incorrectly assume that everything possible is done to relieve pain in their child [12]
• The greatest degree of parental distress is caused by failing to protect their child from pain causes [52, 53]
• ‘Taking care of pain’ is rated as the second highest priority of parents of hospitalised children [54]
• Parents hold misconceptions about how children express their pain [55]
• Parents lack information to make use of pharmacological or behavioural interventions to treat pain at home [11]
• Most parents do not realise that they can play a role in improving pain management for their child [56]

### Advocating for behavioural analgesia

When we think of analgesia, we think of medications. Indeed, we are conditioned to think about analgesic interventions in a binary way: pharmacological and non-pharmacological, valorising pharmacology by defining everything else by its absence. In reality, the most common analgesic interventions are behavioural, where we often avoid pain by moving away from it or its known causes. Procedural pain clashes with our evolved behaviour to avoid pain: in choosing a procedure, we choose pain and offset short-term pain, for example caused by a vaccination, with long-term gain. For adults, this cognitive work is complex and multi-faceted, and decisions can be difficult to sustain. But babies and young children cannot make these decisions. We are choosing pain for our children, and their experience is not tempered by understanding future needs. Given that psychological strategies such as behavioural avoidance or cognitive offsetting are not available to children, we should offer analgesic strategies that are known to be highly effective, such as distraction [28], and seek to reduce anxiety with therapeutic touch, breastfeeding, and optimal positioning [47].

Building on the ‘moments that matter’ concept—which implies that long-term pain beliefs are shaped by key pain experiences—teaching parents how to support their children during painful vaccinations, using distraction, tactile intervention, or breastfeeding could be a key educational moment with long-term beneficial effects. Frequent reasons, for not using these interventional approaches, such as not allowing babies to be breastfed during vaccination because they will ‘choke’ are unfounded [57], yet are all too easily used [58]. History tells us there has been a longstanding ‘denial of infant pain’ [59] and that a lack of pain provision is far too common. It can be easier to convince yourself that it ‘won’t hurt’ rather than accepting that you are doing something that is inducing (potentially unnecessary) pain and is therefore unacceptable. To address this, we need myth busting campaigns on a local, national, and international scale, using high-profile media outlets. Industry can help here—‘See my pain’ campaign [60] has resulted in personal pain assessment tools being made available in more than 2327 general practice sites.

**Fig. 2:**
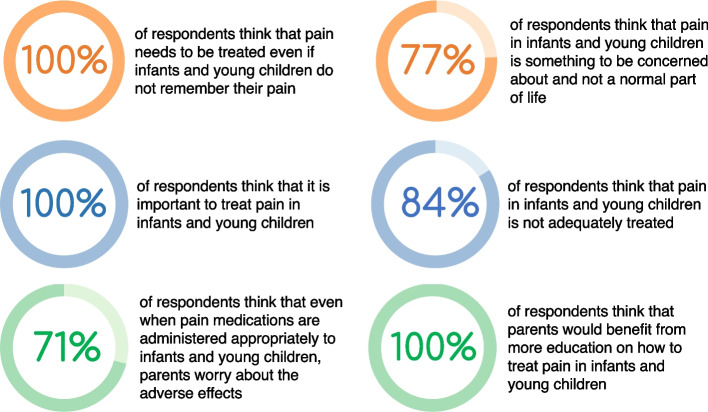
Key findings from a survey of 32 clinicians who have experience working in European Healthcare Settings [61]

An online survey [61], which formed the basis of an expert advisory group meeting about pain in childhood, explored the opinions from 32 clinicians in Europe who specialise in Paediatrics, Neonatology, Psychology, or Clinical Medicine, regarding barriers to pain treatment in infants and young children. The results showed that the majority of surveyed health care professionals think that pain in infants and young children is not adequately treated and that parents would benefit from information on pharmacological, behavioural, and psychological interventions (Fig. 2). A focus on information dissemination in this area is likely to have significant positive impacts on child health.

## Conclusions

### A call to action

This article highlights that with adequate information and motivation to act, families and healthcare professionals can advocate for appropriate pain relief in children (Fig. 1). Importantly, while needle-related pain is an archetypal example of pain under-treatment, the value, relevance, and direct transferability of using similar approaches to manage other types of pain in children, such as everyday pains [62] or post-operative pain [63], is clear. To improve pain treatment in infants and young children, we need to focus on information (understanding treatment options and expectations), emotion (motivation to treat), and advocacy for the child. These can be most effectively delivered through prioritising key ‘moments that matter’, such as children’s early life experiences of iatrogenic pain, so that their future expectations about pain relief are appropriately formed and shaped.

A wide variety of communication channels are needed to maximise change, including high-quality information from trusted sources, social media campaigns to increase awareness, education for healthcare professionals, and more. The *Lancet Child and Adolescent Health* Commission on Delivering transformative action in paediatric pain sets out four goals as a call to action: make pain matter, make pain understood, make pain visible, and make pain better [64]. We need a national conversation on how to achieve these goals, which will require the engagement of healthcare professionals, parents, charities, and industry to make this vision a reality. It is within our grasp to promise to do everything possible to prevent and treat pain caused by vaccinations in every child, every time [24]—let us do it.

## Data Availability

No datasets were generated or analysed during the current study.

## Abbreviations

UK: United Kingdom
WHO: World Health Organisation
